# Prognostic Value of CD11b Expression Level for Acute Myeloid Leukemia Patients: A Meta-Analysis

**DOI:** 10.1371/journal.pone.0135981

**Published:** 2015-08-26

**Authors:** Shuangnian Xu, Xi Li, Jianmin Zhang, Jieping Chen

**Affiliations:** Department of Hematology, Southwest Hospital, Third Military Medical University, 30 Gaotanyan Street, Shapingba District, Chongqing, People’s Republic of China; University of Leicester, UNITED KINGDOM

## Abstract

**Background:**

Study results on the prognostic value of CD11b for acute myeloid leukemia (AML) patients are inconsistent. An up-to-date meta-analysis was conducted to assess the prognostic value of CD11b expression level for AML patients.

**Methods:**

Electronic databases including PubMed, Embase, Cochrane Library, Web of Science and Chinese BioMedical Literature Database (CBM) were searched to identify studies that investigated the association between CD11b expression level and prognosis of AML patients. Pooled hazard ratios (HRs) with 95% confidence intervals (CIs) for overall survival (OS) and disease-free survival (DFS) and pooled odds ratio (OR) with 95% CI for complete remission rate (CRR) were calculated using Revman 5.3 and Stata 11.0.

**Results:**

13 total studies with 2619 patients were included in this meta-analysis. Results of the meta-analysis showed that CD11b positivity was associated with lower CRR (OR = 0.44; 95% CI, 0.25–0.79; p = 0.006) and shorter OS (HR = 0.66; 95% CI, 0.55–0.80; p < 0.0001), but did not affect DFS (HR = 0.67; 95% CI, 0.31–1.48; p = 0.32). Subgroup analysis by ethnicity, cut-off value for CD11b positivity, treatment, subtype and sample preparation method showed no significant interaction between these factors with the prognostic value of CD11b expression level for AML patients. Sensitivity analysis yielded consistent results with the main meta-analysis.

**Conclusion:**

CD11b positivity could predict a poor prognosis for AML patients. Thus, CD11b expression level might be considered a prognostic biomarker for AML patients.

## Introduction

Acute myeloid leukemia (AML) is the most common type of leukemia that affects adults, with a prevalence of 3.8 cases per 10,000 adults rising to 17.9 cases per 10,000 adults aged 65 years and older [1]. It is a heterogeneous clonal disorder of hematopoietic stem/progenitor cell which lose the ability to differentiate normally and to respond to normal regulators of proliferation and apoptosis, results in an accumulation of huge amount of immature blasts with variable degrees of myeloid differentiation in the bone marrow and peripheral blood [2,3]. Cell-cell interaction and cell-matrix interaction between AML cells and different tissue/cells is essential for leukemic engraftment, migration and infiltration [4–8]. These biological process are mediated by specific cell surface receptors [9,10].

Cluster of differentiation 11b (CD11b) is a kind of cell surface receptor that are selectively expressed on leukocytes, which is also named as integrin alpha M (ITGAM), complement component 3 receptor alpha chain (CR3a), macrophage-1 antigen alpha subunit or macrophage receptor 1 alpha subunit (MAC1a). In GENE database of national center for biotechnology information (NCBI), this protein is also named as systemic lupus erythematosus type 6 (SLEB6) or MO1A[11, 12,13]. It is one protein subunit that forms the heterodimeric integrin alpha-M beta-2 molecule with cluster of differentiation 18 (CD18), also named as macrophage-1 antigen or macrophage-1 antigen (Mac-1), complement receptor 3 (CR3)or MO1[11, 12,13]. This protein can participate in cell activation, chemotaxis, cytotoxicity, phagocytosis and regulates interaction of leukemic cells with microenvironment through binding to its ligands, such as inactivated complement component 3b (iC3b), intercellular adhesion molecule (ICAM), fibrinogen, beta-glukanes, coagulation factor X etc.[14–19]. Recently, CD11b is also defined as a marker for myeloid-derived suppressor cells, which is reported to be harnessed by malignant cells to restrain antitumor immunity and to promote malignant expansion or refractoriness to treatment [20–22]. So it is presumable that CD11b may participate in the regulation of biology of malignant AML cells and its expression level may affect the prognosis of AML patients.

Actually, CD11b expression level has been considered as an adverse prognostic factor in AML patients since the 90s [23,24]. AML expressing CD11b was even described as a new leukemic syndrome in 1998[25]. Until now, many studies have demonstrated that CD11b positivity is associated with poor prognosis of AML patients[26,27], but still some other studies yielded conflicting results[28], which means that the prognostic value of CD11b for AML patients is controversial. Therefore, we conducted this up-to-date meta-analysis by combining all published literature to assess the prognostic value of CD11b expression level for AML patients.

## Materials and Methods

This work was carried out following the Cochrane Handbook of systematic reviews and was reported based on PRISMA (Preferred Reporting Items for Systematic Reviews and Meta-Analyses) statement [29].

### Identification of relevant studies

The following electronic databases were systematically searched for relevant studies from inception to July 2015 without language restrictions: PubMed, Embase, Cochrane Library, Web of Science and Chinese BioMedical Literature Database (CBM). The detailed search strategies for each database are reported in S1 Table.

### Study selection

Two authors independently estimated the eligibility of studies by screening the title and abstract of each article identified by above literature search. After excluding obviously irrelevant articles, full-texts were obtained and assessed by the same two authors independently. Disagreements were resolved by consensus.

The inclusion criteria included a) prospective and historical cohort studies; b) studies that evaluated the association between CD11b expression level and the prognosis of AML patients; c) studies that provided sufficient data to estimate hazard ratios (HRs) with 95% confidence intervals (CIs) for overall survival (OS) and disease-free survival (DFS) or odds ratio (OR) with 95% CI for complete remission rate (CRR). When multiple papers reported on the same study, only the most updated one was included.

### Data extraction and quality assessment

Data were carefully extracted from all eligible studies independently by two authors including first author, publication year, region, study design, patients’ characteristics, CD11b detection method and predominant treatment regimen for patients.

Methodological quality was assessed by two authors according to the Newcastle-Ottawa Quality Assessment Scale (NOS) which was based on three categories: selection, comparability, and outcome. The full score was 9 points, and a high-quality study in our analysis was defined as a study with ≥7 points [30]. Any disagreement was resolved by consensus.

### Statistical analysis

For time-to-event data, OS and DFS, the log HRs and their standard errors were directly extracted from the published articles or indirectly calculated from the reported events and the p value in the log-rank test or from the published Kaplan-Meier curves [31, 32]. We pooled the log HRs and corresponding 95% CIs across studies with the generic inverse-variance method and the weight for each study was calculated by the inverse variances of their effect estimates [33]. For dichotomous data, CRR, we extracted events in each arm and calculated OR and corresponding 95% CI. The Mantel-Haenszel method was used to pool ORs and 95% CIs across studies and the weight for each study was calculated on the size of the study and the number of events [34].

Statistical heterogeneity between studies was assessed by χ^2^ based Q test with a significant level at p < 0.1 and quantified with *I* ^*2*^ statistic (*I* ^*2*^ = 0–25%: no heterogeneity; *I* ^*2*^ = 25–50%: moderate heterogeneity; *I* ^*2*^ = 50–75%: large heterogeneity; *I* ^*2*^ = 75–100%: extreme heterogeneity) [35]. Fixed-effect model was chosen for summary estimation if heterogeneity was not significant, whereas random-effects model was adopted if heterogeneity was significant. Subgroup analysis and meta-regression were performed to assess the influence of study region, cut-off value for CD11b positivity, treatment, subtype and sample preparation method on the prognostic value of CD11b expression level in patient with AML. Publication bias was assessed using funnel plots [36].

All analyses were conducted in Review Manager Version 5.3 (Revman, the Cochrane Collaboration, Oxford, England) and Stata version 11.0 (STATA Crop, College Station, Texas). A two-sided p-value of ≤ 0.05 was considered significant for all analyses except heterogeneity tests.

## Results

### Basic characteristics and methodological quality of eligible studies

**Fig 1:**
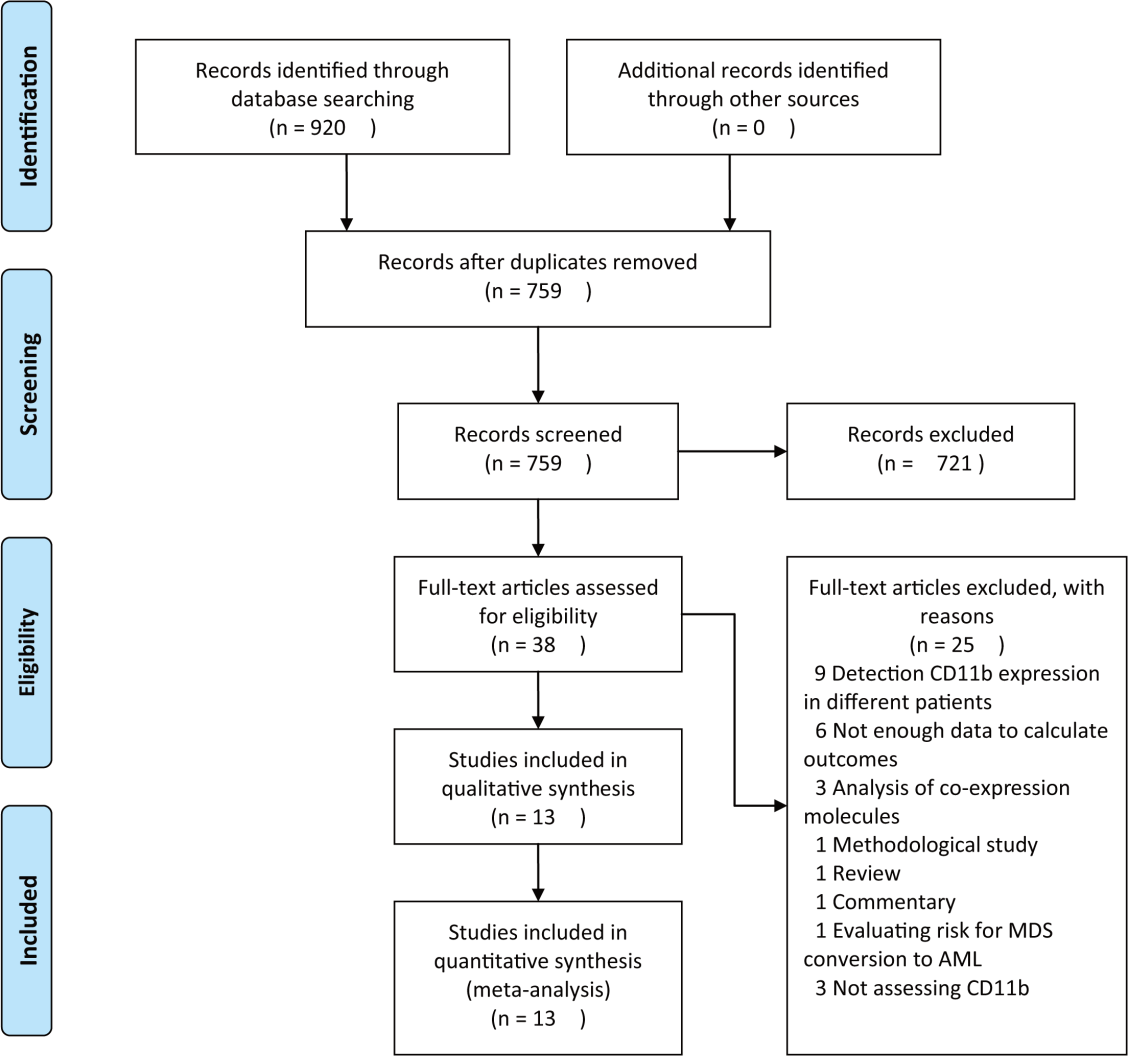
Flow chart of study selection and identification.

The flow chart of literature search and selection was shown in Fig 1. Totally, 917 reports were retrieved and 13 studies with 2619 patients were eligible for the meta-analysis after screening title and abstract and reviewing the full-text articles [23–28, 37–43]. The main characteristics of the included studies are shown in Table 1. All the included studies were aiming to investigate the prognostic value of CD11b expression level for AML patients. 11 studies suggested that CD11b positivity is associated poor prognosis of AML patents [23–27, 37–41, 43], but two studies yielded conflicting results [28, 42]. Among them, 10 studies [24, 25, 27, 28, 38–43] reported results of CRR, five studies [24, 26–28, 38] reported results of OS and three studies [23, 27, 28] reported results of DFS. Nine studies [23, 24, 26, 28, 37–39, 41, 42] are prospective cohort studies and four studies [25, 27, 40, 43] are retrospective cohort studies. Five studies were conducted in western countries [23–25, 27, 38] and eight studies were conducted in eastern countries [26, 28, 37, 39–43]. Patients in ten studies were treated by standard chemotherapy [23–25, 27, 28, 37–39, 42, 43], patients in two studies were treated by hematopoietic stem cell transplantation (HSCT) [26, 41] and the treatment strategy was not reported in one study [40]. Eight studies [23,27,37–39,41–43] defined positivity of CD11b by a cut-off value of 20%, one study [28] defined positivity of CD11b by a cut-off value of 30%, one study [25] defined positivity of CD11b by a cut-off value of 32%, and the cut-off value for CD11b positivity was not available in the other three studies [24,26,40]. Five studies [23, 28, 37, 39, 42] enrolled all AML patients, one study only enrolled AML-M5 patients [41], two studies excluded AML-M3 patients [38, 43], and the subtype information was not available in the other five studies [24–27, 40]. Four studies [23–25, 28] adopted ficoll-hypaque gradient centrifugation (FHGC) as sample preparation method, three studies [27, 38, 42] adopted red blood cell lysis as sample preparation method, and the other five studies did not reported information about sample preparation method [26, 37, 39–41]. Six studies [25,27,38,41–43] reported the equipment used for detection of CD11b positivity, one study [37] adopted varied equipment because different research centers uses different flow cytometers, and the other six studies [25,26,28,37,39,40] did not reported specific information about equipment used. Seven studies [23, 25, 27, 28, 41] reported the source of antibody used, one study [37] adopted varied antibodies because different research centers uses different antibodies, the other six studies [23, 26, 37, 38, 40] did not reported specific information about antibody used. The score of quality assessment ranges from 5 to 9, and the detailed scoring items of the included 13 studies were shown in Table 2.

**Table 1:** Basic characteristics of includes studies.

First author	Albitar et al	Amirghofran et al	Bradstock et al	Chen et al	Chen et al	Junca et al	Liang et al	Paietta et al	Tucker et al	Xu et al	Xu et al	Yang et al	Zhuang et al
Publication year	2011	2001	1994	2013a	2013b	2014	2001	1998	1990	2006	2009	2014	2011
Region	S.A	Iran	Australia	China	Canada	Spain	China	America	UK	China	China	China	China
Study Design	Prospective cohort	Prospective cohort	Prospective cohort	Prospective cohort	Prospective cohort	Retrospective cohort	Prospective cohort	Retrospective cohort	Prospective cohort	Retrospective cohort	Prospective cohort	Prospective cohort	Retrospective cohort
No. of Patients	62	70	120	510	233	158	80	382	92	136	113	516	147
Gender(M/F)	NA	NA	NA	295/215	137/96	78/80	51/29	214/168	53/39	NA	62/51	290/226	76/71
Age(median, range; years)	8 (0.7–14)	32.7(10–70)	(15–60)	36(12–83)	61(18–90)	56(14–78)	37(11–67)	45(15–78)	42(15–65)	Over 18	375(16–68)	17–88	54(15–89)
WBC (median, range), 10^9^/L	NA	61(0.7–650)	NA	NA	5(0–606)	NA	NA	NA	33(0–235)	NA	NA	NA	NA
Sample type	NA	PB or BM	PB or BM	BM	PB or BM	PB or BM	BM	PB or BM	PB	NA	BM	BM	BM
Sample preparation method	NA	FHGC	FHGC	NA	red blood cell lysis	red blood cell lysis	NA	FHGC	FHGC	NA	NA	red blood cell lysis	NA
Detection method	FL	FL or IF	FL	FL	FL	FL	APAAP	FL	FL	FL	FL	FL	FL
Equipment	NA	NA	Varied*	NA	EPICS XL-MCL	EPICS XL-MCL	NA	NA	Coulter Epics C	NA	FACS Calibur	FACSCanto II	FACS Calibur
Source of antibody	NA	Dako	Varied*	NA	NA	Beckman Coulter	BD	BD	NA	NA	ebioscience	BD	BD
Cut off value	NA	30%	NA	20%	20%	20%	20%	32%	20%	NA	20%	20%	20%
Dynamic range	NA	NA	NA	NA	NA	NA	NA	NA	NA	NA	NA	NA	NA
CD11b+ patients(cases, percent)	25(40%)	44(62.9%)	40(33%)	23%	145(70%)	53(36%)	24(30%)	95(25%)	48(52%)	71(52.2%)	83(73.45)	123(23.8%)	65(44.2%)
FAB type													
M0	NA	0	NA	0	23	NA	0	NA	0	NA	0	10	0
M1	NA	12	NA	26	37	NA	11	NA	31	NA	0	34	3
M2	NA	12	NA	147	44	NA	21	NA	19	NA	0	146	107
M3	NA	9	NA	78	0	NA	15	NA	5	NA	0	111	0
M4	NA	30	NA	60	22	NA	13	NA	19	NA	0	49	7
M5	NA	4	NA	109	25	NA	18	NA	14	NA	113	139	20
M6	NA	3	NA	10	4	NA	2	NA	3	NA	0	26	9
M7	NA	0	NA	5	7	NA	0	NA	0	NA	0	1	1
Unidentified	NA	NA	NA	75	71	NA	0	NA	0	NA	0	0	0
Cytogenetics													
Favorable	0	NA	NA	NA	0	21	NA	NA	NA	NA	NA	NA	NA
Intermediate	46	NA	NA	NA	0	97	NA	NA	NA	NA	NA	NA	NA
Unfavorable	16	NA	NA	NA	233	33	NA	NA	NA	NA	NA	NA	NA
Treatment (predominant)	HSCT	Standard CT	Standard CT	Standard CT	Standard CT	Standard CT	Standard CT	Standard CT	Standard CT	NA	HSCT	Standard CT	Standard CT

APAAP = alkaline phosphatase-anti-alkaline phosphatase complex method, HSCT = Hematopoietic stem cell transplantation, S.A = Saudi Arabia, CT = chemotherapy, NA = DATA not available, FHGC = ficoll-hypaque gradient centrifugation, BD = Becton Dickinson.

* means the equipment or antibody varied between different research centers in this study.

**Table 2:** The assessment of the risk of bias in each cohort study using the Newcastle-ottawa scale.

Study	Selection				Comparability		Outcome			Total
	REC	SNEC	AE	DO	SC	AF	AO	FU	AFU	
Albitar 2011	*	*	-	*	-	-	*	-	*	5
Amirghofran 2001	*	*	*	*	-	-	*	*	*	7
Bradstock 1994	*	*	-	*	-	-	*	*	*	6
Chen 2013a	*	*	*	*	-	-	*	*	*	7
Chen 2013b	*	*	*	*	-	-	*	*	*	7
Junca 2014	*	*	*	*	-	-	*	*	*	7
Liang 2001	*	*	*	*	*	*	*	*	*	9
Paietta 1998p	*	*	*	*	-	-	*	*	*	7
Tucker 1990	*	*	*	*	-	-	*	*	*	7
Xu 2006	*	*	-	*	-	-	*	*	*	6
Xu 2009	*	*	*	*	*	-	*	*	*	8
Yang 2014	*	*	*	*	-	-	*	*	*	7
Zhang 2011	*	*	*	*	-	-	*	*	*	7

REC = representativeness of the exposed cohort, SNEC = selection of the nonexposed cohort, AE = ascertainment of exposure, DO = demonstration that outcome of interest was not present at start of study, SC = study controls for age, subtype, AF = study controls for white blood cell number at diagnosis and treatment, AO = assessment of outcome, FU = follow-up long enough for outcomes to occur (for studies that only assessed CR, ‘long enough’ is defined as 6 month, for studies that assessed survival data, ‘long enough’ is defined as 3 years), AFU = adequacy of follow-up of cohorts (≥80%).

“*” means that the study is satisfied the item and “-” means the opposite situation.

### CD11b expression level and CRR of AML patients

**Fig 2:**
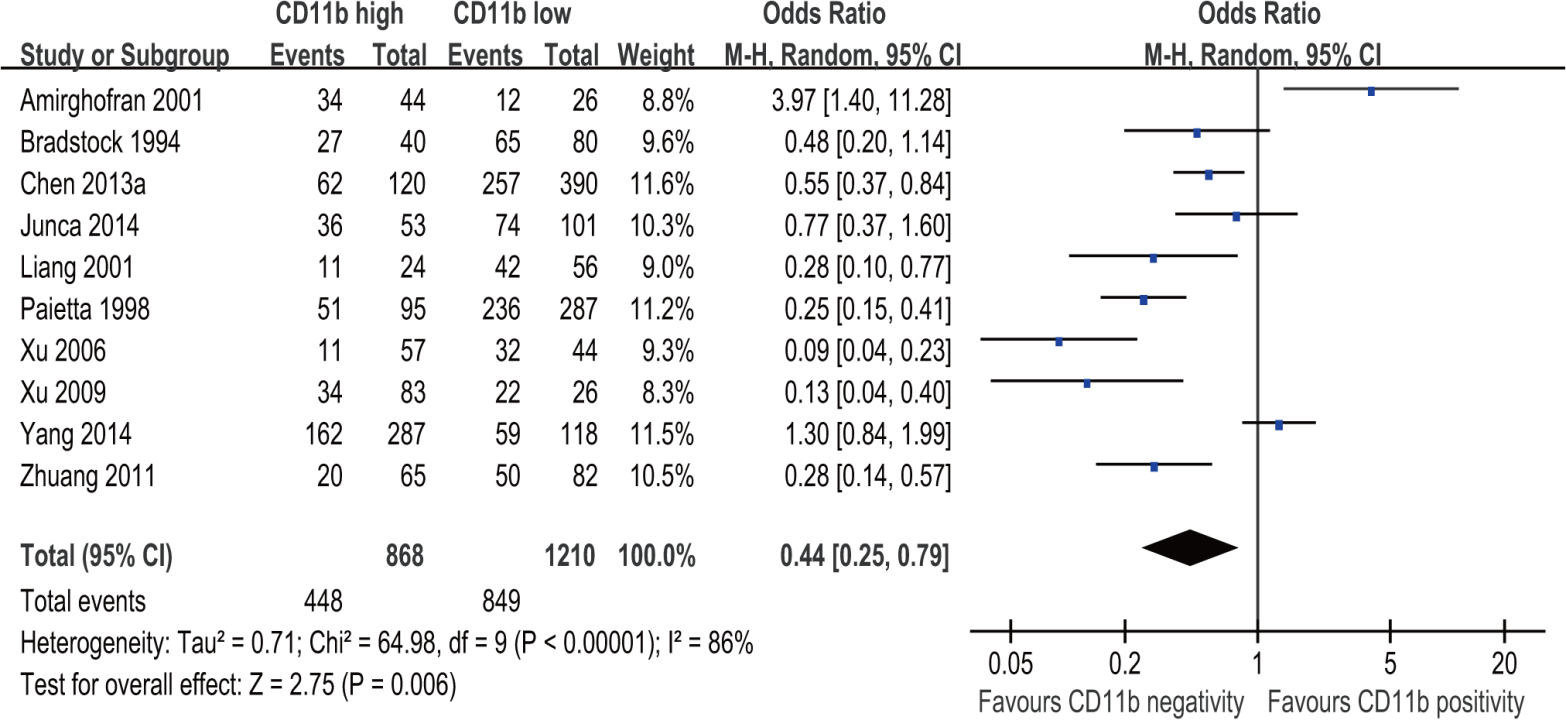
Forest plot for the association between CD11b expression level and complete remission rate (CRR) of AML patients.

10 studies with 2078 patients assessed the association of CD11b expression level with CRR in AML. The event in each group is defined as acquirement of complete remission for AML patients. The result of meta-analysis for CRR showed that patients with CD11b positivity had a significantly decreased CRR compared with patients with CD11b negativity (OR = 0.44; 95% CI, 0.25–0.79; p = 0.006; Fig 2) although with significant heterogeneity among the studies (I^2^ = 86%; p < 0.00001).

Subgroup analysis showed no significant interaction between the CRR effect of CD11b expression with study country, cut-off value for CD11b positivity, treatment, subtype and sample preparation method (Table 3).

**Table 3:** Summary of subgroup analysis results for CD11b and prognosis of AML patients.

Subgroup		Sample size	Effect measures		Heterogeneity		Meta-regression
			HR/OR (95% CI)	p-value	I^2^(%)	p-value	p-value
			CR				
Country	Western	656	0.43(0.21, 0.89)	0.02	70	0.04	0.98
	Eastern	1422	0.44 (0.20, 0.98)	0.04	89	<0.0001	
Cut-off value	20%	1475	0.60 (0.31, 1.15)	0.12	84	<0.0001	0.10
	32%	382	0.25 (0.15, 0.41)	<0.0001	NA	NA	
	NA	221	0.25 (0.04, 1.08)	0.01	86	<0.0001	
Treatment	HSCT	109	0.13 (0.04, 0.40)	0.0004	NA	NA	0.04
	Standard CT	1969	0.50(0.28,0.90)	0.02	86	<0.0001	
Subtype	AML as a whole	1822	0.53 (0.28, 1.02)	0.06	87	<0.0001	0.08
	AML without M3	147	0.28 (0.14, 0.57)	0.0003	NA	NA	
	AML-M5	109	0.13 (0.04, 0.40)	0.0004	NA	NA	
Sample preparation method	FHGC	572	0.74(0.16,3.35)	0.70	91	<0.0001	0.19
	red blood cell lysis	706	0.68(0.27,1.68)	0.40	85	0.001	
	NA	800	0.22(0.08, 0.57)	0.002	81	0.001	
			OS				
Country	Western	511	0.71 (0.58, 0.87)	0.001	0	0.71	0.05
	Eastern	132	0.33 (0.22, 0.68)	0.001	0	1.00	
Cut-off value	20%	391	0.73 (0.56, 0.95)	0.02	0	0.43	0.32
	30%	70	0.39 (0.18, 0.86)	0.02	NA	NA	
	NA	182	0.64 (0.48, 0.86)	0.003	40	0.20	
Treatment	HSCT	62	0.39 (0.17, 0.87)	0.02	NA	NA	0.18
	Standard CT	581	0.69 (0.56, 0.83)	0.0002	0	0.42	
Subtype	AML as a whole	410	0.66 (0.53, 0.83)	0.0005	35	0.21	0.95
	AML without M3	233	0.67 (0.48, 0.94)	0.02	NA	NA	
Sample preparation method	FHGC	190	0.64 (0.48, 0.35)	0.003	43	0.19	0.33
	red blood cell lysis	391	0.73 (0.56, 0.95)	0.02	0	0.43	
	NA	62	0.39 (0.17, 0.87)	0.01	NA	NA	

95% CI = 95% confidence interval, DFS = disease-free survival, HR = hazard ratio, NA = data not available, OR = odds ratio, OS = overall survival.

### CD11b expression level and OS of AML patients

**Fig 3:**
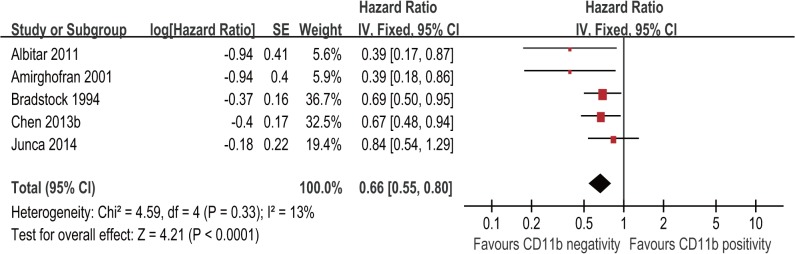
Forest plot for the association between CD11b expression level and overall survival (OS) of AML patients.

Five studies with 643 patients assessed the association of CD11b expression level with OS in AML. The result of meta-analysis for OS showed that patients with CD11b positivity had a significantly shorter OS compared with patients with CD11b negativity (HR = 0.66; 95% CI, 0.55–0.80; p < 0.00001; Fig 3) with no significant heterogeneity among the studies (I^2^ = 13%; p = 0.33).

Subgroup analysis showed no significant interaction between the OS effect of CD11b expression with study country, cut-off value for CD11b positivity, treatment, subtype and sample preparation method (Table 3).

### CD11b expression level and DFS of AML patients

**Fig 4:**
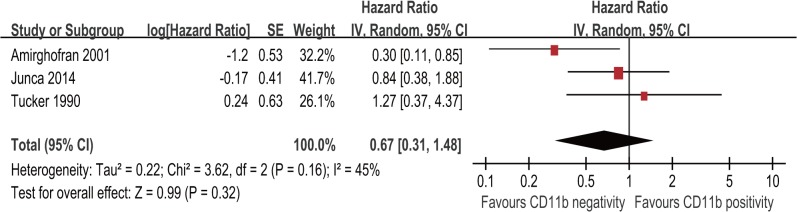
Forest plot for the association between CD11b expression level and disease-free survival (DFS) of AML patients.

Three studies with 320 patients assessed the association of CD11b expression level with DFS in AML. The result of meta-analysis for DFS showed that patients with CD11b positivity had a similar DFS compared with patients with CD11b negativity (HR = 0.67; 95% CI, 0.31–1.48; p = 0.32, Fig 4) with no significant heterogeneity among the studies (I^2^ = 45%; p = 0.16). Since only three studies were included in this meta-analysis, subgroup analysis was not conducted.

### Sensitivity analysis and publication bias

**Fig 5:**
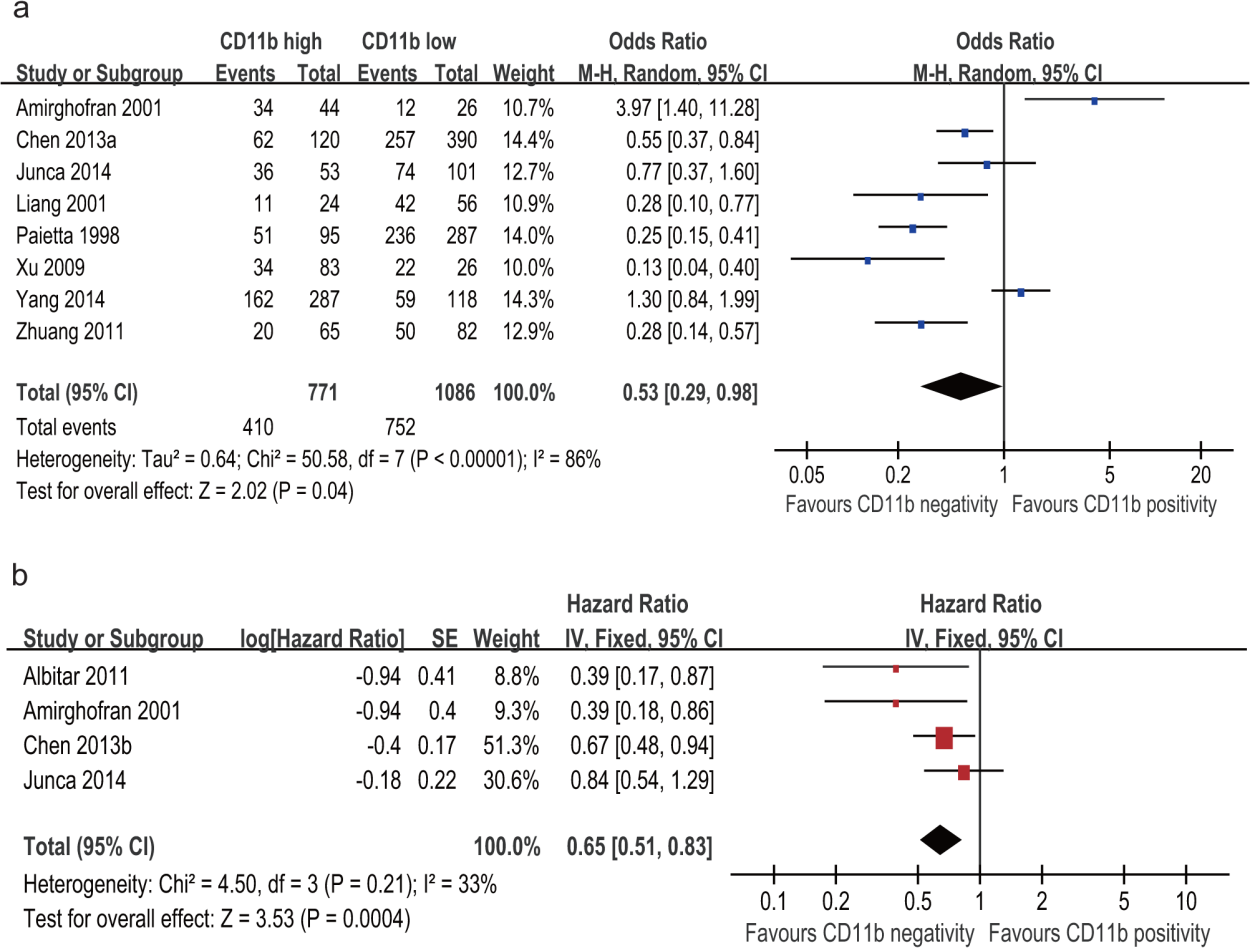
Forest plot for sensitivity analysis by only including high quality score studies for the association between CD11b expression level and CRR of AML patients (a) and for the association between CD11b expression level and OS of AML patients (b).

A sensitivity analysis for CRR and OS was conducted by only including high NOS score studies to assess the effect of study quality on the stability of this meta-analysis, the results of sensitivity analysis is consistent with the main meta-analysis, suggesting that the results of this meta-analysis is reliable (Fig 5). Since all studies included in the meta-analysis for DFS are with high quality, so we didn’t perform this sensitivity analysis for this outcome.

Another sensitivity analysis, in which one study was removed at a time, was also conducted. The pooled HRs or ORs were not significantly changed, further indicating the stability of our analyses (Table 4).

**Table 4:** Sensitivity analysis by omitting each of the included studies in different outcomes.

Outcomes	Omitted Study	HR or OR	95% CI	P	I^2^(%)	Ph
CR	Amirghofran 2001	0.36	0.21–0.63	0.0003	84%	<0.00001
	Bradstock 1994	0.44	0.23–0.83	0.01	88%	<0.00001
	Chen 2013a	0.43	0.21–0.86	0.02	88%	<0.00001
	Junca 2014	0.42	0.22–0.79	0.007	87%	<0.00001
	Liang 2001	0.46	0.25–0.86	0.02	87%	<0.00001
	Paietta 1998	0.48	0.26–0.89	0.02	86%	<0.00001
	Xu 2006	0.52	0.30–0.92	0.02	84%	<0.00001
	Xu 2009	0.47	0.25–0.88	0.02	87%	<0.00001
	Yang 2014	0.39	0.22–0.68	0.001	81%	<0.00001
	Zhang 2011	0.50	0.28–0.90	0.02	86%	<0.00001
OS	Albitar 2011	0.69	0.56–0.83	0.0002	0%	0.42
	Amirghofran 2001	0.69	0.57–0.84	0.0002	0%	0.44
	Bradstock 1994	0.65	0.51–0.83	0.004	33%	0.21
	Chen 2013b	0.66	0.53–0.83	0.0005	35%	0.21
	Junca 2014	0.63	0.51–0.78	<0.0001	8%	0.35
DFS	Amirghofran 2001	0.95	0.49–1.87	0.89	0%	0.59
	Junca 2014	0.59	0.15–2.43	0.47	67%	0.08
	Tucker 1990	0.53	0.20–1.45	0.22	58%	0.12

CRR = complete remission rate, DFS = disease free survival, HR = hazard ratio, Ph = p for heterogeneity, OR = odds ratio, OS = overall survival.

**Fig 6:**
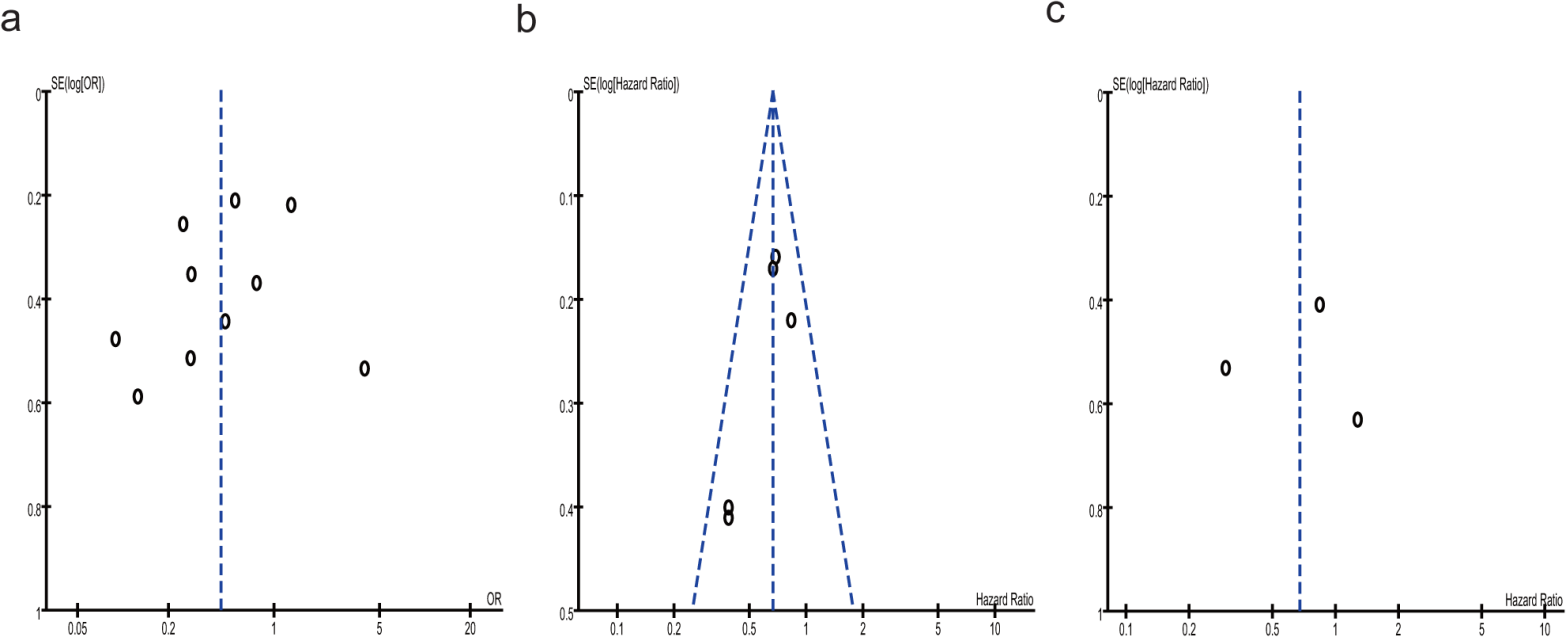
The funnel plots were largely symmetric suggesting there were no publication biases in the meta-analysis of CD11b expression level and prognosis of AML patients. The funnel plot from ten studies assessed the association between CD11b expression level and CRR of AML patients (a). The funnel plot from five studies assessed the association between CD11b expression level and OS of AML patients (b). The funnel plot from three studies assessing the association between CD11b expression level and DFS of AML patients (c).

The funnel plots were largely symmetric suggesting that there were no publication biases in this meta-analysis of CD11b expression level and prognosis of AML patients (Fig 6).

## Discussion

Although CD11b expression level has long been recognized with prognostic value for AML patients, the results are controversial between different studies. This may be attributed to the statistical limitation (e.g., small sample size) of individual study, different ethnicity of included participants, different antibody or equipment used or varied cut-off value for CD11b positivity. Thus, we performed this meta-analysis with subgroup analysis and sensitivity analysis to pool these relevant studies together to resolve this controversial issue and provide up-to-date clinical evidence for adopting CD11b expression level as a prognostic biomarker for AML patients.

To the best to our knowledge, this is the first meta-analysis that evaluates the role of CD11b expression level for predicting the prognosis of AML patients. Results of our meta-analysis showed that compared with AML patients with CD11b negativity, AML patients with CD11b positivity are associated with lower CRR, shorter OS, but has no significant effect on DFS.

Previous studies evaluating the prognostic role of CD11b expression level in AML patients have enrolled participants with different ethnicity and different subtypes, adopted varied cut-off value for CD11b positivity ranging from 20% to 32% and conducted different treatment for recruited participants. Thus, we undertook subgroup analyses according to these factors to investigate the interaction between these factors with the results of this meta-analysis. We also conducted sensitivity analyses by only including high quality score studies and by omitting each study. Results of different subgroup or sensitivity analyses are consistent with the main meta-analyses, indicating the results of this meta-analysis are reliable. Taken together, these results clearly demonstrated that CD11b expression level might be regarded as a prognostic biomarker for AML patients.

CD11b is a protein subunit of integrin alpha-M beta-2 molecule which is essential for cell-cell interaction between leukemic cells with its microenvironment [8, 10], and then participates in regulation of biological activities of leukemic cells [13–19]. Currently, CD11b is also defined as a marker for myeloid-derived suppressor cells, which is reported to be involved in restraining antitumor immunity of the host and promoting expansion and drug-resistance of hematological malignant cells [20, 22, 44]. So it is mechanistically reasonable that CD11b expression level should be regarded as a prognostic biomarker for AML patients.

Meta-analysis of large amount of patients can provide direct and definite evidence for assessing the prognostic biomarkers for AML patients. This meta-analysis integrated the data from different clinical studies evaluating the prognostic value of CD11b expression level for AML patients in different countries for the first time, hence the statistical power is increased and the applicability is widened. What is more, most of the included cohort studies are with high quality and no statistically significant publication bias for each outcome was noted which also ensure reliability of this meta-analysis. Last but not the least, although sample preparation method, equipment and antibody used for detection of CD11b varied between studies, results of subgroup analysis according to sample preparation method showed no significant interaction between these factors with results, which suggests that the prognostic value of CD11b expression level is valid.

However, there are some limitations of this meta-analysis. Firstly, this meta-analysis is based on summary data rather than individual patients’ data, although we have undertaken subgroup analysis trying to evaluate the prognostic value of CD11b expression level in different subgroup of patients, but we could not explore more detailed or even patient-level prognostic value of CD11b expression level. Secondly, different length of follow-up among included studies might affect the evaluation of this meta-analysis. Thirdly, heterogeneity cannot be avoided in certain analysis which forced us to use the relatively conservative random effect model in these conditions. Last, the meta-analysis for DFS only included three studies, so this result should be interpreted with caution.

In conclusion, besides the limitations mentioned above, our meta-analysis indicates that CD11b expression level is closely related to the prognosis of AML patients and should be considered as a prognostic biomarker for stratifying AML patients. It might be also promising to develop drugs that target CD11b for improving the prognosis of AML patients.

## Supporting Information

S1 Table Search strategies for PubMed, Embase, Cochrane Library, Web of Science and Chinese BioMedical Literature Database. (DOCX)

S2 Table PRISMA Checklist for the Systematic Review and Meta-analysis to Estimate the prognostic value of CD11b expression level for AML patients. (DOC)
