# Supplementary material for: Prognostic Value of CD11b Expression Level for Acute Myeloid Leukemia Patients: A Meta-Analysis
Source: PLoS One. 2015 Aug 26;10(8):e0135981. doi: 10.1371/journal.pone.0135981 (PMC4550244; doi:10.1371/journal.pone.0135981)
Supplement: S1 Table — (DOCX) [file pone.0135981.s001.docx]

**Supplementary Table 1**

**Part 1.** PubMed search strategy

**Part 2.** Embase search strategy

**Part 3.** Cochrane Library search strategy

**Part 4.** Web of Science search strategy

**Part 5.** Chinese BioMedical Literature Database search strategy

**Part 1. PubMed search strategy**

(((((progno*[Title/Abstract]) OR survival[Title/Abstract]) OR outcome[Title/Abstract])) AND (((((((((((((((acute myeloid leukemia[Title/Abstract]) OR acute myelogenous leukemia[Title/Abstract]) OR acute granulocytic leukemia[Title/Abstract]) OR acute myeloid leukaemia[Title/Abstract]) OR acute myelogenous leukaemia[Title/Abstract]) OR acute granulocytic leukaemia[Title/Abstract]) OR acute promyelocytic leukemia[Title/Abstract]) OR acute promyelocytic leukemia[Title/Abstract]) OR acute promyelocytic leukemia[Title/Abstract]) OR acute myeloblastic leukemia[Title/Abstract]) OR acute myeloblastic leukaemia[Title/Abstract]) OR acute non lymphoblastic leukemia[Title/Abstract]) OR AML[Title/Abstract]) OR APL[Title/Abstract]) OR ANLL[Title/Abstract])) AND (((((((((((((((((CD11b[Title/Abstract]) OR cluster of differentiation 11b[Title/Abstract] OR ITGAM[Title/Abstract]) OR integrin, alpha M[Title/Abstract]) OR complement component 3 receptor alpha chain[Title/Abstract]) OR CR3A[Title/Abstract]) OR macrophage receptor 1 alpha subunit [Title/Abstract]) OR macrophage-1 antigen alpha subunit [Title/Abstract]) OR MAC1A[Title/Abstract]) OR MO1A[Title/Abstract]) OR integrin alpha-M beta-2 [Title/Abstract]) OR macrophage receptor 1 [Title/Abstract]) OR macrophage-1 antigen [Title/Abstract]) OR MAC-1 [Title/Abstract]) OR complement receptor 3 [Title/Abstract]) OR CR3 [Title/Abstract]) OR MO1[Title/Abstract]) OR Systemic lupus erythematosus type 6 [Title/Abstract]) OR SLEB6[Title/Abstract])

**Part 2.** **Embase search strategy**

#1 acute myeloid leukemia: ab,ti

#2 acute myelogenous leukemia: ab,ti

#3 acute granulocytic leukemia: ab,ti

#4 acute myeloid leukaemia: ab,ti

#5 acute myelogenous leukaemia: ab,ti

#6 acute granulocytic leukaemia: ab,ti

#7 acute promyelocytic leukemia: ab,ti

#8 acute promyelocytic leukaemia: ab,ti

#9 acute myeloblastic leukemia: ab,ti

#10 acute myeloblastic leukaemia: ab,ti

#11 acute nonlymphoblastic leukemia: ab,ti

#12 acute nonlymphoblastic leukaemia: ab,ti

#13 AML: ab,ti

#14 APL: ab,ti

#15 ANLL: ab,ti

#16 #1 or #2 or #3 or #4 or #5 or #6 or #7 or #8 or #9 or #10 or #11 or #12 or #13 or #14 or #15

#17 ‘cluster of differentiation 11b’ : ab,ti

#18 cd11b: ab,ti

#19 ‘Integrin alpha M’: ab,ti

#20 ITGAM: ab,ti

#21 ‘complement component 3 receptor alpha chain’: ab,ti

#22 CR3A: ab,ti

#22 ‘macrophage receptor 1 alpha subunit’: ab,ti

#24 ‘macrophage-1 antigen alpha subunit’: ab,ti

#25 MAC1A: ab,ti

#26 MO1A: ab,ti

#27 ‘integrin alpha-M beta-2’: ab,ti

#28 ‘macrophage receptor 1’: ab,ti

#29 ‘macrophage-1 antigen’: ab,ti

#30 MAC-1: ab,ti

#31 ‘complement receptor 3’: ab,ti

#32 CR3: ab,ti

#33 MO1: ab,ti

#34 ‘Systemic lupus erythematosus type 6’: ab,ti

#35 SLEB6: ab,ti

#36 #17 or #18 or #19 or #20 or #21 or #22 or #23 or #24 or #25

#37 progno*: ab,ti

#38 survival: ab,ti

#39 outcome: ab,ti

#40 #27 or #28 or #29

#41 #16 AND #26 AND #30

**Part 3. Cochrane Library search strategy**

#1 cluster of differentiation 11b

#2 CD11b

#3 Integrin alpha M

#4 ITGAM

#5 complement component 3 receptor alpha chain

#6 CR3A

#7 macrophage receptor 1 alpha subunit

#8 macrophage-1 antigen alpha subunit

#9 MAC1A

#10 MO1A

#11 integrin alpha-M beta-2

#12 macrophage receptor 1

#13 macrophage-1 antigen

#14 MAC-1

#15 complement receptor 3

#16 CR3

#17 MO1

#18 Systemic lupus erythematosus type 6

#19 SLEB6

#20 #1 or #2 or #3 or #5 or #6 or #7 or #8 or #9 or #11 or #12 or #13 or #14 or #15 or #16 or #17 or #18 or #19

#21 acute myeloid leukemia

#22 acute myelogenous leukemia

#23 acute granulocytic leukemia

#24 acute myeloid leukaemia

#25 acute myelogenous leukaemia

#26 acute granulocytic leukaemia

#27 acute promyelocytic leukemia

#28 acute promyelocytic leukaemia

#29 acute myeloblastic leukemia: ab,ti

#30 acute myeloblastic leukaemia: ab,ti

#31 acute non-lymphoblastic leukemia

#32 acute non lymphoblastic leukemia

#33 AML

#34 APL

#35 ANLL

#36 #21 or #22 or #23 or #24 or #25 or #26 or #27 or #28 or #29 or #30 or #31 or #32 or #33

#37 progno*

#38 survival

#39 outcome

#40 #37 or #38 or #39

#41 #20 and #36 and #40

**Part 4 Web of Science search strategy**

#1 TOPIC: (progn*)

#2 TOPIC: (survival)

#3 TOPIC: (outcome)

#4 #1 OR #2 OR #3

#5 TOPIC: (cluster of differentiation 11b)

#6 TOPIC: (CD11b)

#7 TOPIC: (integrin, alpha M)

#8 TOPIC: (ITGAM)

#9 TOPIC: (complement component 3 receptor alpha chain)

#10 TOPIC: (CR3A)

#11 TOPIC: (macrophage receptor 1 alpha subunit)

#12 TOPIC: (macrophage-1 antigen alpha subunit)

#13 TOPIC: (MAC1A)

#14 TOPIC: (MO1A)

#15 TOPIC: (integrin alpha-M beta-2)

#16 TOPIC: (macrophage receptor 1)

#17 TOPIC: (macrophage-1 antigen)

#18 TOPIC: (MAC-1)

#19 TOPIC: (complement receptor 3)

#20 TOPIC: (CR3)

#21 TOPIC: (MO1)

#22 TOPIC: (Systemic lupus erythematosus type 6)

#23 TOPIC: (SLEB6)

#24 #5 OR #6 OR #7 OR #8 OR #9 OR #10 OR #11 OR #12 OR #13 OR #14 OR #15 OR #16 OR #17 OR #18 OR #19 OR #20 OR #21 OR #22 OR #23

#25 TOPIC: (acute myeloid leukemia)

#26 TOPIC: (acute myelogenous leukemia)

#27 TOPIC: (acute granulocytic leukemia)

#28 TOPIC: (acute myeloid leukaemia)

#29 TOPIC: (acute myelogenous leukaemia)

#30 TOPIC: (acute granulocytic leukaemia)

#31 TOPIC: (acute promyelocytic leukemia)

#32 TOPIC: (acute promyelocytic leukaemia)

#33 TOPIC: (acute myeloblastic leukemia)

#34 TOPIC: (acute myeloblastic leukaemia)

#35 TOPIC: (acute non-lymphoblastic leukemia)

#36 TOPIC: (acute non lymphoblastic leukemia)

#37 TOPIC: (AML)

#38 TOPIC: (APL)

#39 TOPIC: (ANLL)

#40 #25 OR #26 OR #27 OR #28 OR #29 OR #30 OR #31 OR #32 OR #33 OR #34 OR #35 OR #36 OR #37

#41 #4 AND #24 AND #40

**Part 5. Chinese BioMedical Literature Database search strategy**

#1 cluster of differentiation 11b AND leukemia

#2 cd11b and leukemia

#3 integrin alpha m and leukemia

#4 ITGAM and leukemia

#5 complement component 3 receptor alpha chain and leukemia

#6 cr3a and leukemia

#7 macrophage receptor 1 alpha subunit and leukemia

#8 macrophage-1 antigen alpha subunit and leukemia

#9 mac1a and leukemia

#10 mo1a and leukemia

#11 integrin alpha-m beta-2 and leukemia

#12 macrophage receptor 1 and leukemia

#13 macrophage-1 antigen and leukemia

#14 mac-1 and leukemia

#15 complement receptor 3 and leukemia

#16 cr3 and leukemia

#17 mo1 and leukemia

#18 systemic lupus erythematosus type 6 and leukemia

#19 sleb6 and leukemia

#20 All literature retrieved by each search term were imported into Endnote
